# The complete chloroplast genome of *Miliusa glochidioides* (Annonaceae) and phylogenetic analysis

**DOI:** 10.1080/23802359.2022.2116947

**Published:** 2022-09-05

**Authors:** Yangying Gan, Qi Zhang, Jingyao Ping

**Affiliations:** a Key Laboratory of Urban Agriculture in South China, Ministry of Agriculture and Rural Affairs, Institute of Agricultural Economics and Information, Guangdong Academy of Agricultural Sciences, Guangzhou, China; b Guangdong Provincial Key Laboratory for Crop Germplasm Resources Preservation and Utilization, Agro-biological Gene Research Center, Guangdong Academy of Agricultural Sciences, Guangzhou, China; c College of Life Sciences, South China Agricultural University, Guangzhou, China

**Keywords:** *Miliusa glochidioides*, chloroplast genome, phylogenetic analysis

## Abstract

The chloroplast (cp) genome of *Miliusa glochidioides* has been fully sequenced. The cp genome of this species has a typical quadripartite structure comprised of four parts: a large single copy (LSC; 88,782 bp) region, a small single copy (SSC; 18,949 bp) region, and two inverted repeat (IR; 26,029 bp each) regions. The full length of the cp genome is 159,789 bp; its GC content is 36.7%, and it encodes a total of 129 genes including 84 protein-coding genes, 37 tRNA genes, and 8 rRNA genes. Among the protein-coding genes, nine (*rps16*, *rpl2*, *rpl16*, *atpF*, *rpoC1*, *petB*, *petD*, *ndhA,* and *ndhB*) contain one intron, and three (*rps*12, *clpP,* and *ycf*3) have two introns. A maximum-likelihood (ML) phylogenetic tree shows that *M. glochidioides* is a sister to *Chieniodendron hainanense.*

*Miliusa glochidioides* Handel-Mazzetti 1933 is an evergreen shrub with a height of 1.5 meters that belong to family Annonaceae, and is distributed on forested slopes below 900 meters in Guangxi, China, where it is endemic. *Miliusa* plants are often used as ropes or kindling due to their strong and slender stems (Li et al. 2011). In the Annonaceae Checklist (http://herbarium.botanik.univie.ac.at/annonaceae/listTax.php), this species is treated as a synonym of *Orophea polycarpa* (Rainer and Chatrou 2006). However, serious discrepancies exist in the descriptions of these two taxa within China (Li et al. 2011). Obtaining the complete chloroplast (cp) genome may provide a molecular basis for investigating phylogenetic relationships and population variation.

Fresh leaves were collected from the South China Botanical Garden of the Chinese Academy of Sciences (E113°36′, N23°18′). The specimens were stored in the Herbarium of South China Agricultural University (Jingyao Ping, email: pingjnyao@foxmail.com) under specimen code PJY-GXYDH2110 (herbaria acronyms follow Thiers, 2021, continuously updated). Total DNA was extracted using the E.Z.N.A.^®^ Plant DNA Kit (OMEGA, Shanghai). After the genomic DNA extracted from a sample was tested for quality, the DNA was broken through physical methods (ultrasonication), and then purified to construct a sequencing library. The steps of this process are as follows: DNA end repair, addition of A to the 3′ end, ligation of sequencing adapters, recovery of the target fragment through agarose gel electrophoresis, polymerase chain reaction amplification of the target fragment, and finally, construction of a sequence library. The Illumina NovaSeq6000 platform was used for paired-end sequencing with a 150-bp read length. The sequences were spliced using the stitching software NOVOPlasty V4.2 (https://github.com/ndierckx/novoplasty) (Dierckxsens et al. 2017), and GetOrganelle V1.7.0 + (https://github.com/Kinggerm/GetOrganelle) (Jin et al. 2020) was used for identification of the optimal assembly results. The assembled cp genome was annotated using PGA (Qu et al. 2019) and GeSeq (Tillich et al. 2017) software. The program MAFFT v7.311 (Katoh and Standley 2013) was used for multiple sequence alignment of the complete cp genome of *M. glochidioides* with those of other eight plants downloaded from GenBank. RAxML v.8.0 software (Stamatakis 2014) was used to construct an maximum-likelihood (ML) tree with 1000 bootstrap replicates using the GTRGAMMAI substitution model.

The cp genome of *M. glochidioides* (GenBank accession number: OM047203) has a typical quadripartite structure with a total length of 159,789 bp and GC content of 39.2%. The lengths of LSC, SSC and IR are 88,782, 18,949 and 26,029 bp, respectively. This cp genome encodes a total of 129 genes, including 84 protein-coding genes, 37 tRNA, and eight rRNA genes. Among the protein-coding genes, nine (*rps16*, *rpl2*, *rpl16*, *atpF*, *rpoC1*, *petB*, *petD*, *ndhA* and *ndhB*) contain one intron and three (*rps12*, *clpP* and *ycf3*) contain two introns. The ML tree strongly supports *M. glochidioides* and *Chieniodendron hainanense* as sister groups (Figure 1). *M. glochidioides* is the first species of its genus whose cp genome has been sequenced, providing reliable information for phylogenetic and cp genomics studies of Annonaceae.

**Figure 1.**
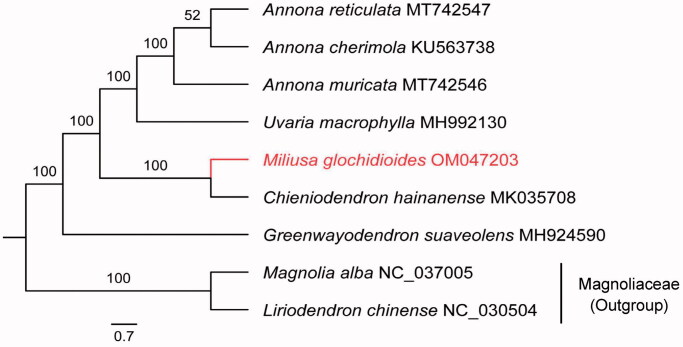
Maximum-likelihood tree based on the sequences of nine complete chloroplast genomes. *Magnolia alba* and *Liriodendron chinense* were selected as outgroup. Numbers in the nodes were bootstrap values from 1000 replicates.

## Data Availability

The genome sequence data that support the findings of this study are available in GenBank of NCBI at [https://www.ncbi.nlm.nih.gov] under the accession no. MT742546, MT742547, MK035708, MH924590, MH992130, KU563738, NC_037005, NC_030504, and OM047203. These data were derived from the following resources available in the public domain: [https://www.ncbi.nlm.nih.gov/nuccore/] The associated BioProject, SRA, and Bio-Sample numbers are PRJNA793237, SRR17406795, SAMN24519632, respectively.
